# Dose‐dependent effects of kefir on colitis induced by trinitrobenzene sulfonic acid in rats

**DOI:** 10.1002/fsn3.1174

**Published:** 2019-08-22

**Authors:** Nurhayat Ozkan Sevencan, Mehmet Isler, Fatma Nilgun Kapucuoglu, Altug Senol, Burcak Kayhan, Sefa Kiztanir, Muhammed Cem Kockar

**Affiliations:** ^1^ Department of Internal Medicine, Medical Faculty The University of Karabuk Karabuk Turkey; ^2^ Department of Internal Medicine, Medical Faculty The University of Suleyman Demirel Isparta Turkey; ^3^ Department of Gastroenterology, Medical Faculty The University of Suleyman Demirel Isparta Turkey; ^4^ Department of Gastroenterology Davraz Yasam Hospital Isparta Turkey; ^5^ Department of Pathology, Medical Faculty The University of Suleyman Demirel Isparta Turkey; ^6^ Department of Pathology, Medical Faculty The University of Koc Istanbul Turkey

**Keywords:** colitis, inflammatory bowel disease, kefir, probiotic, trinitrobenzene sulfonic acid

## Abstract

Evidence suggests that gut microbiota dysbiosis plays a critical role in the initiation and promotion of inflammatory bowel disease (IBD). Kefir is a fermented dairy product including yeast and bacterial species. We aimed to investigate the effect of kefir on trinitrobenzene sulfonic acid (TNBS)‐induced colitis in rats using two different doses. Fifty‐four Wistar rats were divided into six groups. For 14 days, the normal control and colitis control groups were given tap water, kefir10 control, kefir10 colitis, and kefir30 control, and the kefir30 colitis groups were given phosphate‐buffered saline containing 10% or 30% kefir, respectively, instead of tap water. Colitis was induced by intracolonically administrating TNBS in the colitis control, kefir10 colitis, and kefir30 colitis groups. On the 14th day, the rats were sacrificed. The weights and lengths of the colons were measured and macroscopically evaluated, and the distal 10 cm segments were subjected to a histopathological examination. The incidence of bloody stool and diarrhea in the kefir10 colitis group was found to be less than the colitis control and kefir30 colitis groups. The colonic weight/length ratio in the kefir10 colitis group was lower than that in the colitis control and kefir30 colitis groups. We detected that the 10% kefir treatment reduced TNBS‐induced macroscopic colonic damage, while it was exacerbated by the 30% kefir treatment. No significant difference was observed between the colitis groups in terms of microscopic colonic damage scoring. These results indicate that kefir, with a careful dose selection, may be a useful agent in the treatment of IBD.

## 1 INTRODUCTION

It is widely accepted that genetic factors, immune disorders, and environmental factors play a role in the pathogenesis of inflammatory bowel disease (IBD) (Strober, Fuss, & Mannon, 2007). Currently, anti‐inflammatory and immunosuppressive drugs, antibiotics, and biological agents used in the treatment of IBD are useful in varying degrees to achieve and maintain remission, but none of them are curative.

Recent studies have strongly suggested that gut microbiota dysbiosis initiates and promotes the inflammatory process (Chassaing & Gewirtz, 2014; Munyaka, Eissa, Bernstein, Khafipour, & Ghia, 2015). Despite the large variation present in an individual's gut microbiota, the majority of species belong to the Firmicutes, Bacteroidetes, Proteobacteria, and Actinobacteria phyla (De Cruz et al., 2015; Rigottier‐Gois, 2013). It has been found that in individuals with IBD, many taxa, including Firmicutes and Bacteroidetes, have decreased (Foligne et al., 2007; Frank et al., 2007), and microbial diversity has been shown to be lower in patients with ulcerative colitis (Sha et al., 2013) and Crohn's disease (Quince et al., 2015; Sha et al., 2013) than in healthy controls.

Therefore, in the treatment of IBD, the use of probiotics for modulating the impaired intestinal flora is extensively investigated. Probiotics are “live microorganisms that, when administered in adequate amounts, confer a health benefit on the host” (Hill et al., 2014). A meta‐analysis evaluating the efficacy of probiotics in IBD revealed that probiotics may be as effective as 5‐ASA in preventing quiescent ulcerative colitis (UC) relapse. VSL #3, a probiotic preparation that contains eight different types of bacteria, may be effective in inducing remission of active ulcerative colitis. However, the efficacy of probiotics in Crohn's disease remains unclear (Derwa, Gracie, Hamlin, & Ford, 2017).

Kefir is a natural probiotic beverage based on fermented milk, for which various health‐promoting properties are attributed (Bourrie, Willing, & Cotter, 2016; Marsh, O'Sullivan, Hill, Ross, & Cotter, 2013). It is a dynamic fermented dairy product including yeast and bacterial species, as well as metabolites, such as exopolysaccharides (Bourrie et al., 2016). An analysis of 25 kefir milk and associated grains sourced from eight geographically distinct regions using high‐through put sequencing showed that the bacterial populations in kefir were dominated by two phyla, the Firmicutes and the Proteobacteria (Marsh et al., 2013). Many previous studies have shown that kefir and kefir fractions have anti‐inflammatory and antioxidant activity (Bourrie et al., 2016), and the ameliorating effect of kefir on DSS‐induced colitis in rats was determined in one study (Senol et al., 2015).

The aim of this study is to describe the effects of kefir with different two doses in trinitrobenzene sulfonic acid (TNBS)‐induced colitis in rats.

## 2 MATERIALS AND METHODS

### 2.1 Experimental animals

Fifty‐four 18‐week‐old Wistar albino male rats weighing between 200 and 250 g were used. The animals were kept in a temperature‐ and illumination‐controlled room (22–24°C; 12 hr each of light and darkness) over the course of study. They were placed separately in individual cages. Wire‐mesh floors were laid in the cages to prevent coprophagia.

All the animals were fed with standard laboratory chow ad libitum. The rats were not fed on the 6th day of the study (24 hr prior to induction of colitis), but fluid intake was not restricted. The rats continued to be fed after the induction of colitis. Some rats were given tap water as drinking water, and some were given phosphate‐buffered saline (PBS) containing 10% or 30% kefir, ad libitum. The PBS containing kefir was renewed every 12 hr.

The amounts of chow, tap water, or PBS consumed by each rat were recorded every 12 hr, whereas the rats' body weights were monitored daily. The rat stools were monitored daily, and the presence of diarrhea and bloody stools was recorded.

### 2.2 Kefir

A commercial kefir starter culture containing *Lactobacillus lactis* subs., *Leuconostoc* subs., *Streptococcus thermophilus, Lactobacillus* subs., and yeast of kefir (Danisco^®^) was used in this study. Milk containing 3% fat was heated to 25°C, and after the addition of 3% kefir starter culture, the lids of the jars were closed and incubated at 37°C for 12 hr. The final product was put into a plastic container and stored at +4°C. Kefir was prepared on a daily basis over the course of study.

### 2.3 Experimental groups

As shown in Table 1, the rats were randomized to six groups: normal control, kefir10 control, kefir30 control, colitis control, kefir10 colitis, and kefir30 colitis. The normal control and colitis control groups drank tap water for 14 days. For 14 days, kefir10 control and kefir10 colitis groups were given PBS containing 10% kefir instead of tap water, while the kefir30 control and kefir30 colitis groups were given PBS containing 30% kefir.

**Table 1:** Experimental groups

Group	*n*	Rectal application (Day 7)	Drinking fluid (Days 1–14)
Normal control	8	Physiological serum	Tap water
Kefir10 control	8	Physiological serum	PBS containing 10% kefir
Kefir30 control	8	Physiological serum	PBS containing 30% kefir
Colitis control	10	TNBS	Tap water
Kefir10 colitis	10	TNBS	PBS containing 10% kefir
Kefir30 colitis	10	TNBS	PBS containing 30% kefir

Abbreviations: PBS, phosphate‐buffered saline; TNBS, Trinitrobenzene sulfonic acid.

### 2.4 Induction of colitis

For the rats in the colitis control, kefir10 colitis, and kefir30 colitis groups, TNBS colitis was induced on day 7 in accordance with the description of Morris et al. (1989). Briefly, after 24 hr of fasting, the rats were anesthetized with intraperitoneal ketamine (80 mg/kg) + xylazine (10 mg/kg). Then, they were given 0.25 ml of 60 mg/ml TNBS (Sigma) that was dissolved in 50% ethanol by means of a polyurethane catheter inserted 8 cm into the colon via the anus. The rats in the normal control, kefir10 control, and kefir30 control groups were administered physiological saline instead of TNBS. The animals were maintained in a head‐down position for about 1 min to prevent expulsion of the TNBS.

### 2.5 Macroscopic evaluation of colitis

On the 7th day of intrarectal TNBS or saline administration, between 9 and 10 a.m., the rats were anesthetized by intraperitoneal ketamine hydrochloride (80 mg/kg) and xylazine (10 mg/kg). The rats were then weighed and sacrificed by cervical dislocation. The abdomens of the rats were opened by a midline incision, and their colons were removed. The colons were longitudinally dissected, examined in terms of the presence of diarrheic stool in the lumen, and then washed with saline and blotted. The weight, length, and wall thickness of the colon were measured. Macroscopic colonic inflammation and damage was evaluated according to the Wallace criteria (Wallace & Keenan, 1990). In brief, the grading scale consisted of four criteria: the presence of hyperemia or ulcer (normal appearance, focal hyperemia, and the number and length of ulceration; 0–10), the presence of adhesions (absent, minor, major; 0–2), diarrhea (absent, present; 0 or 1), and colon thickness (mm). A macroscopic evaluation of colitis was done by an independent observer who was uninformed about the treatments performed in rats. After the macroscopic evaluation of the colon, the distal 10 cm segment was separated for histopathological examination.

### 2.6 Histopathologic examination

Histopathologic examinations were performed by a blinded, unbiased pathologist. Colon specimens from the rats were fixed with 10% formaldehyde. Paraffin blocks were prepared, and sections of 5 μm in thickness were obtained from them. Slides were prepared, stained with hematoxylin and eosin (H&E), and examined under a light microscope (with ×100, ×200 and ×400 magnification). A histopathologic evaluation was performed according to the criteria presented in Table 2 (Maximum score: 27. From Peran et al. (2005)).

**Table 2:** Criteria for assessment of microscopic colonic damage

Mucosal epithelium
Ulceration: none (0); mild—surface (1); moderate (2); extensive‐full thickness (3)
Crypts
Mitotic activity: lower third (0); mild mid‐third (1); moderate mid‐third (2); upper third (3)
Mucus depletion: none (0); mild (1); moderate (2); severe (3)
Lamina propria
Mononuclear infiltrate: none (0); mild (1); moderate (2); severe (3)
Granulocyte infiltrate: none (0); mild (1); moderate (2); severe (3)
Vascularity: none (0); mild (1); moderate (2); severe (3)
Submucosal
Mononuclear infiltrate: none (0); mild (1); moderate (2); severe (3)
Granulocyte infiltrate: none (0); mild (1); moderate (2); severe (3)
Edema: none (0); mild (1); moderate (2); severe (3)

Note

Maximum score: 27.

### 2.7 Statistical analysis

A Kolmogorov–Smirnov test was performed to determine whether the distribution of data was normal. For the groups exhibiting normal distribution, a one‐way ANOVA test was used for the comparison of the groups of three or more, and Bonferroni correction was selected as a post hoc adjustment. For the groups that did not exhibit normal distribution, a Kruskal–Wallis test was implemented for a comparison of the groups of three or more, and a Mann–Whitney U test was used for a comparison of the two groups. All the analyses were carried out by IBM Statistical Package for Social Sciences version 24.0 (IBM Corporation). A two‐tailed *p* value < .05 was accepted to be statistically significant.

## 3 RESULTS

### 3.1 Clinical follow‐up

#### 3.1.1 The effect of kefir treatment on the incidence of diarrhea and bloody stools

**Figure 1:**
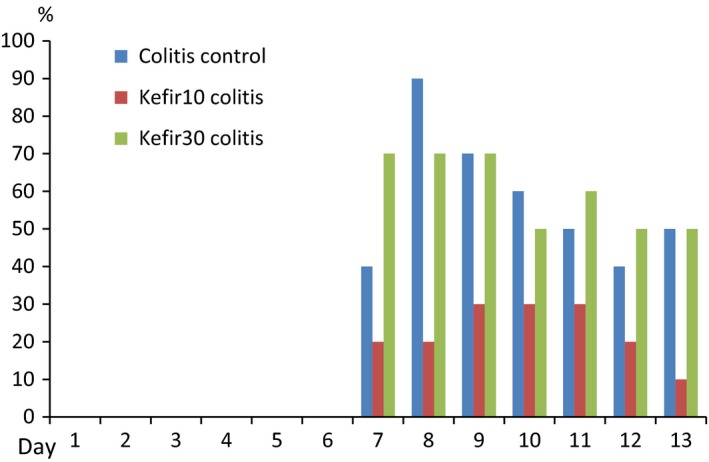
Diarrhea rates in colitis groups

No diarrhea or bloody stool was observed in the noncolitis control groups during the study. In the colitis groups, normal stools were observed in the days before TNBS administration. On day 1 following the administration of TNBS, in the colitis control, kefir10 colitis, and kefir30 colitis groups, bloody stools were noted in 80%, 50%, and 90% of the rats, respectively. Kefir10 treatment also reduced the incidence of diarrhea. Diarrhea was observed in 40% of the rats in the colitis control group, 20% in the kefir10 colitis group, and 70% in the kefir30 colitis group following the first day of colitis induction. On day 7 following TNBS administration, diarrhea was detected in 50%, 10%, and 50%, respectively, of the rats in these groups (Figure 1).

#### 3.1.2 Fluid and chow consumption of rats

**Figure 2:**
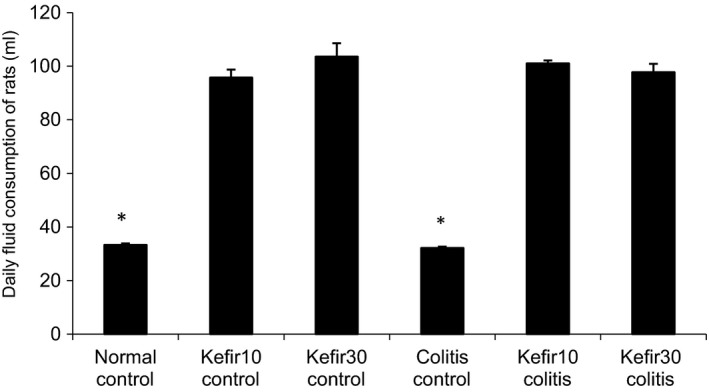
Daily average fluid amounts consumed by experimental groups throughout the study. *According to kefir10 control, kefir30 control, kefir10 colitis, kefir30 colitis groups *p* < .001

Since rats normally drink 30–45 ml of water per day, we predicted that when we added kefir at a concentration of 10% or 30% into a PBS solution as drinking water, a rat would receive approximately 3 to 4.5 ml or 9 to 13.5 ml of kefir per day, respectively. However, interestingly, rats receiving PBS with kefir as drinking water consumed about three times more fluid than expected. The rats in the normal control and colitis control groups consumed 33.3 ± 0.6 and 32.2 ± 0.5 ml of tap water daily, whereas daily fluid consumption was around 100 ml in the rats treated with kefir (Figure 2). Thus, the rats were given 10% or 30% concentration of kefir in PBS actually received an average of 10 or 30 ml of kefir per day, respectively.

No significant difference was seen in chow consumption between the groups before colitis induction. Intracolonic administration of TNBS caused anorexia in the early days. Chow consumption decreased significantly in the colitis control, kefir10 colitis, and kefir30 colitis groups compared to the control groups in the 24 hr following the induction of colitis (*p* < .001). On the second day of colitis induction, the amount of chow consumed in kefir10 colitis rats was somewhat lower than in the control groups, but not statistically different. In the colitis control and kefir30 colitis groups, chow consumption increased to pre‐TNBS levels 4 and 6 days after the induction of colitis, respectively (data not shown).

**Figure 3:**
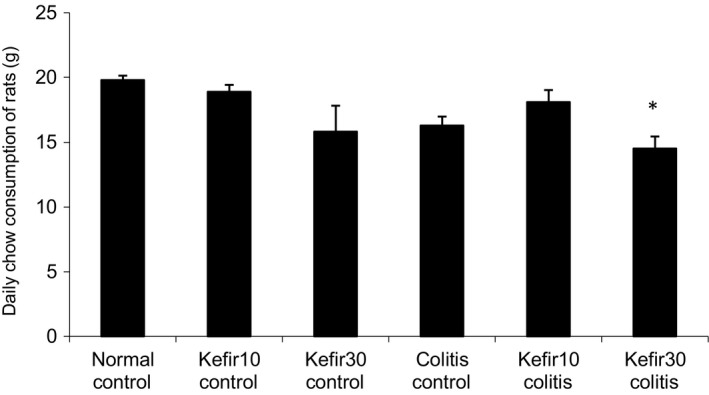
Average daily chow consumption of rat groups throughout the experiment. *According to normal control *p* = .01

The average daily chow amounts consumed during the entire study were evaluated, and daily chow consumption among all groups was the lowest in rats in the kefir30 colitis group. The daily chow consumption of rats in the kefir10 colitis group was close to that in the normal control and kefir10 control groups. The mean daily chow consumption of the rats in the kefir30 control group was found to be lower than the normal control, kefir10 control, and even colitis control and kefir10 colitis groups, but not statistically significant (Figure 3).

#### 3.1.3 Daily body weight changes of rats

No significant difference was noted between the groups in terms of the daily weights of rats until colitis induction. Colitis induction caused a decrease in the body weight of rats; however, the reduction in body weight of rats in the kefir10 colitis group was never statistically significant. On the first day following the induction of colitis, the body weight of rats in the colitis control group was significantly lower than in the normal control group (*p* = .001). On the 2nd and 3rd days following induction, the body weights of the rats were significantly lower in the colitis control and kefir30 colitis groups than in the normal control group (*p* < .01, *p* < .05, respectively). On day 4, the body weight of the rats in the colitis control group was still significantly lower than in the normal control group (*p* < .05). The body weights of animals from day 5 were not significantly different between groups (data not shown).

### 3.2 The effect of kefir on colon weight, length, and weight/length ratio

Colon length, weight measurements, and macroscopic evaluations of the removed colons were done by researchers who were unaware of the rat groups. As shown in Table 3, the mean colon weight in the colitis control group was significantly higher than in the normal control and kefir30 control groups (*p* < .05, *p* < .05). In addition, the weight of the colon in the kefir30 colitis group was significantly higher than that of the normal control, kefir10 control, and kefir30 control groups (respectively, *p* < .01, *p* < .05, *p* < .01).

**Table 3:** The colon weight, length, and weight/length ratio

Groups	*n*	Colon weight (g)	Colon length (cm)	Colon weight/length ratio
(1) Normal control	8	2.3 (2.14–2.55)^a^	15.8 (13–17.3)	0.15 (0.13–0.17)
		2.33 ± 0.05^b^	15.44 ± 0.61	0.15 ± 0.005
(2) Kefir10 control	8	2.4 (2–2.94)	16.3 (13.3–7.5)	0.15 (0.12–0.17)
		2.42 ± 0.13	15.90 ± 0.55	0.15 ± 0.005
(3) Kefir30 control	8	2.4 (1.68–2.84)	15.2 (13–20.5)	0.16 (0.11–0.18)
		2.30 ± 0.13	15.46 ± 0.92	0.15 ± 0.01
(4) Colitis control	10	2.7 (2.22–3.75)	14.5 (12–17.5)	0.20 (0.14–0.26)
		2.96 ± 0.18	14.42 ± 0.49	0.21 ± 0.01
(5) Kefir10 colitis	10	2.7 (2.21–2.98)	13.7 (11.2–6.5)	0.18 (0.15–0.25)
		2.61 ± 0.08	13.80 ± 0.49	0.19 ± 0.01
(6) Kefir30 colitis	10	2.9 (2.47–4.23)	13 (10.7–16)	0.23 (0.16–0.31)
		3.11 ± 0.20	13.19 ± 0.44	0.24 ± 0.01
		4 versus 1, 3*	6 versus 2*	4 versus 1, 2, 3**
		6 versus 2*		5 versus 1, 2**
		6 versus 1, 3**		5 versus 3, 6*
				6 versus 1, 2, 3***

a Median (min‐max);

b Mean ± *SEM*.

* *p* < .05;

** *p* < .01;

*** *p* < .001.

The colon height of the rats in the kefir30 colitis group was significantly shorter than those in the kefir10 control group (*p* < .05).

The colon weight/length ratio was significantly higher in the colitis control group than in the normal control, kefir10 control, and kefir30 control groups (all, *p* < .01). The colon weight/length ratio in the kefir10 colitis group was found to be significantly higher than in the normal control (*p* < .01), kefir10 control (*p* < .01), and kefir30 control (*p* < .05) groups. The colon weight/length ratio in the kefir30 colitis group was significantly higher than in the normal control, kefir10 control, kefir30 control, and kefir10 colitis groups (respectively, *p* < .001, <.001, <.001, and <.05).

### 3.3 Macroscopic colonic damage scores

As shown in Table 4, no macroscopic colonic damage was found in the normal control, kefir10 control, and kefir30 control groups. We observed that TNBS‐induced macroscopic colonic damage was partially reduced by treatment with kefir10 but worsened with kefir30 treatment, but this was not significant.

**Table 4:** Macroscopic and microscopic colonic damage scores

Groups	*N*	Macroscopic score	Microscopic score
(1) Normal control	8	0	1.00 (0–4)
		0	1.75 ± 0.49
(2) Kefir10 control	8	0	0.00 (0–3)
		0	0.63 ± 0.38
(3) Kefir30 control	8	0	2.00 (1–4)
		0	2.00 ± 0.38
(4) Colitis control	10	3.00 (2–11)^a^	12.5 (7–18)
		5.50 ± 1.24 b	12.1 ± 1.16
(5) Kefir10 colitis	10	3.00 (1–7)	15.0 (5–17)
		3.30 ± 0.60	12.8 ± 1.38
(6) Kefir30 colitis	10	7.5 (2–14)	12.5 (4–18)
		7.40 ± 1.60	12.0 ± 1.33
		4 versus 1, 2, 3***	2 versus 3*
		5 versus 1, 2, 3***	4 versus 1, 2, 3***
		6 versus 1, 2, 3***	5 versus 1, 2, 3***
			6 versus 1, 2, 3***

a Median (min‐max);

b Mean ± *SEM*.

* *p* < .05;

*** *p* < .001.

### 3.4 Histopathological evaluation

**Figure 4:**
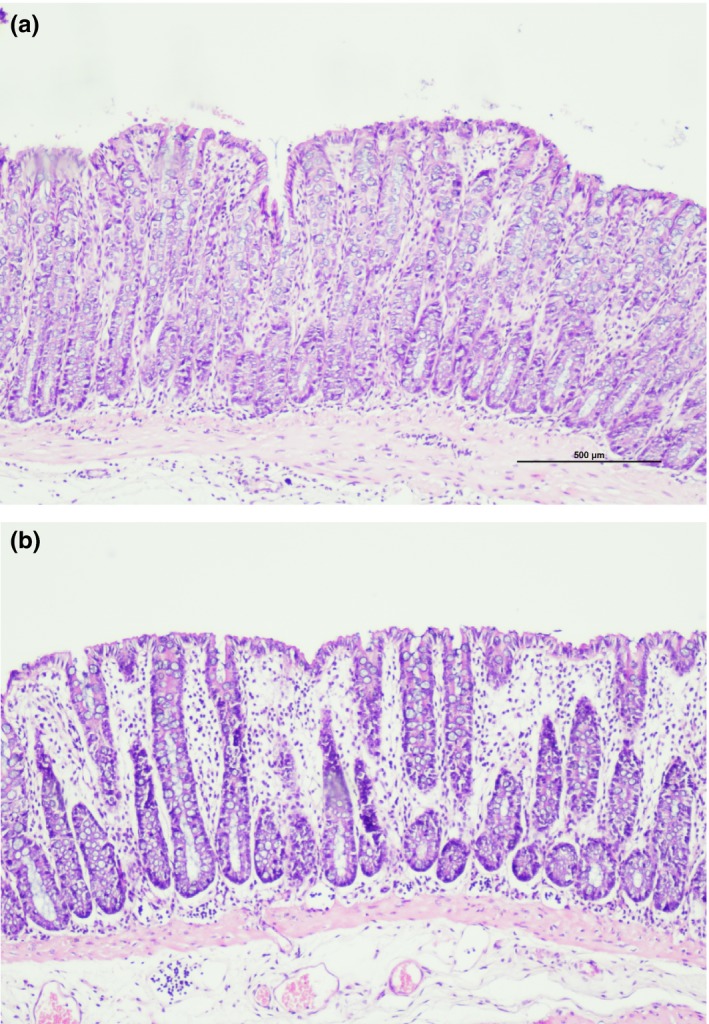
(a) Normal colon mucosa (from normal control group; x10, H&E). (b) Edema in mucosa (from kefir30 control group; x10, H&E)

Among the noncolitis control groups, the microscopic damage score was lowest in the kefir10 control group and highest in the kefir30 control group (Table 4). A histological evaluation of colonic specimens showed no specific finding in the normal control group except focal edema. No significant finding was observed in the kefir10 control group (Figure 4a), except a lymphocyte increase in the lamina propria in one sample. In the kefir30 control group, edema in the lamina propria was observed in all samples (Figure 4b), and moderate edema was seen in the submucosa in some samples.

**Figure 5:**
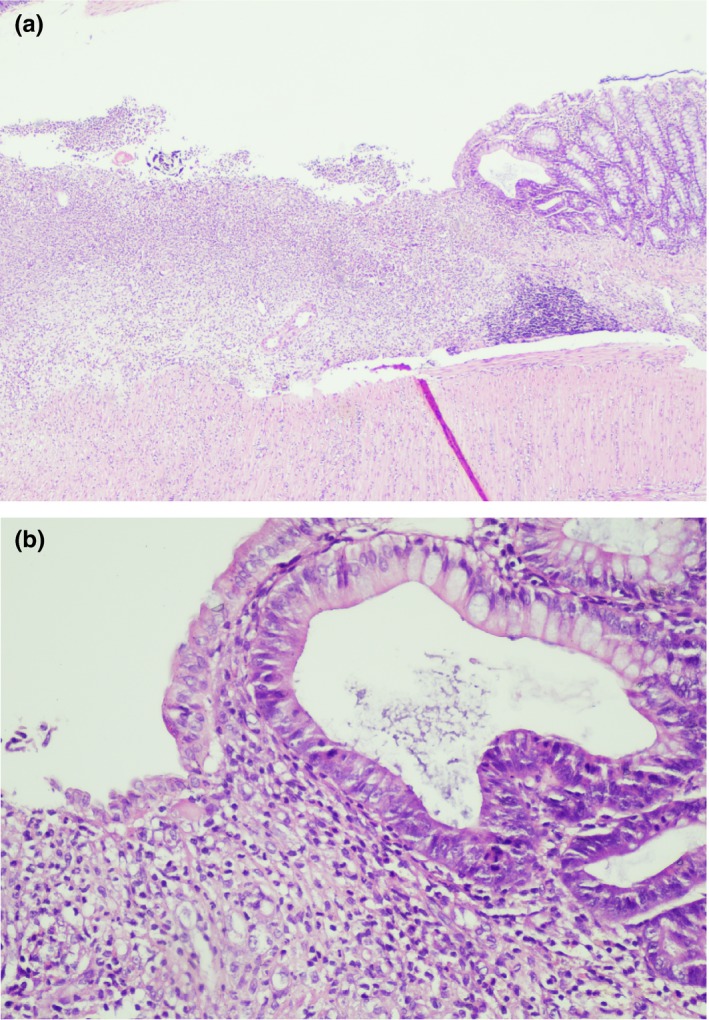
(a) Superficial ulceration (from colitis control group; x4; H&E). (b) Severe mucus depletion, mitosis in the middle third of the crypts (arrow shows mitotic figure, from colitis control group; X20; H&E)

No significant difference was shown between the colitis groups in terms of microscopic colonic damage scoring. In the colitis control group, most of the samples had full‐thickness or superficial ulcers (Figure 5a). All samples had mucin loss in the epithelium, and in nine cases, mitosis increased at different levels in the crypts (Figure 5b). In all but one, a polymorphonuclear leukocyte increased in the lamina propria and was detected from submucosal edema in all subjects.

**Figure 6:**
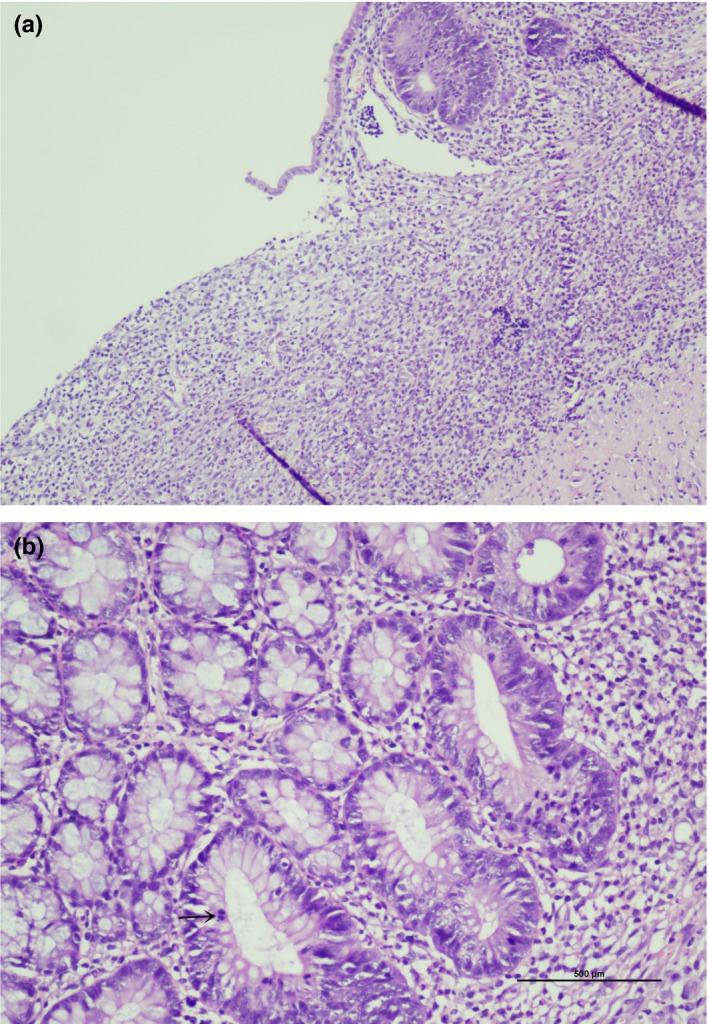
(a) Superficial ulcer (from kefir10 colitis group; X4; H&E). (b) Mild mucus depletion, mitosis in the lower thirds of crypts (arrow shows mitotic figure, from kefir10 colitis group; X20; H&E)

In the kefir10 colitis group, six cases showed ulcers (Figure 6a). All but one had a moderate or significant loss of mucus in the epithelium and increased mitosis in crypts (Figure 6b). All the specimens showed significant edema, polymorphonuclear leukocytes, and mononuclear cell infiltration in the submucosa.

**Figure 7:**
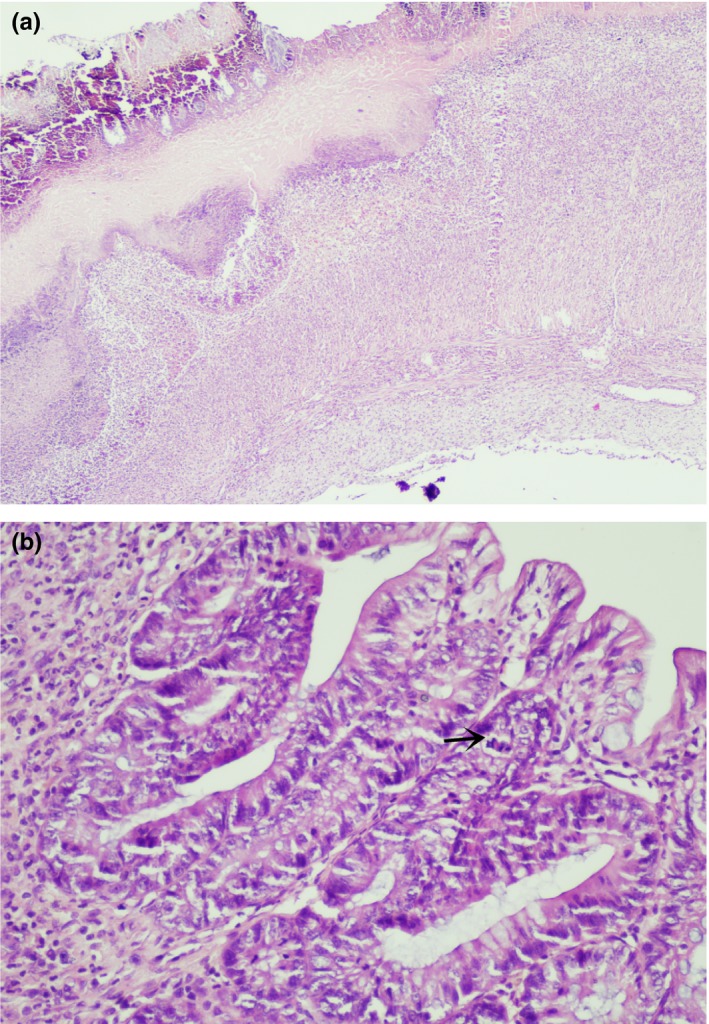
(a) Full‐thickness ulceration (from kefir30 colitis group; X4; H&E). (b) Moderate mucin depletion and mitosis in the upper thirds of the crypts (arrow shows mitotic figure, from kefir30 colitis group; X20; H&E)

In most of the samples in the kefir30 colitis group, ulcers, mucin loss, and an increase in mitosis in cripts were observed (Figure 7a,b). Submucosal edema, polymorphonuclear leukocyte, and mononuclear cell infiltration were more prominent than that of the kefir10 colitis group.

## 4 DISCUSSION

Kefir is a delicious drink that contains abundant fermentation products, such as organic acids and multiple volatile flavor compounds, including ethanol, acetaldehyde, and diacetyl (Güzel‐Seydim, Seydim, Greene, & Bodine, 2000). In the current study, we observed that rats love to drink kefir. Rats were given kefir in PBS as a drinking fluid consumed three times more fluid than those who drink tap water. As a result, rats receiving 10% or 30% kefir in the PBS received an average of 10 or 30 ml kefir per day.

Rectal administration of TNBS elicits a Th1‐mediated immune response and causes transmural colitis characterized by severe mucosal necrosis, macroscopic inflammation, and histological and biochemical intestinal changes, showing similarities with human Crohn's disease (Neurath, Fuss, Kelsall, Stüber, & Strober, 1995). Previous studies generally have shown that whole kefir, microorganisms isolated from kefir, or kefir fractions cause the immune response to shift from Th1 to Th2. Lactobacilli isolated from kefir have been shown to suppress the production of pro‐inflammatory cytokines while increasing anti‐inflammatory cytokine production (Carasi et al., 2015; Hong, Chen, Chen, & Chen, 2009).

Besides the microbial population of the kefir, fermentation products and by‐products of the metabolism of these microorganisms have been shown to have an immunomodulatory effect. Vinderola, Perdigon, et al. (2006) determined that when kefir supernatant was administered, the early increases of the pro‐inflammatory cytokines IL‐1 alpha and TNF alpha were rapidly downregulated by day 7 to values similar to those of the controls by the increase of the regulatory IL‐10 in the adherent cells derived from Peyer's patches (Vinderola, Perdigon, et al., 2006). The increase of the IL‐1 alpha, IFN gamma, and TNF alpha produced by adherent cells derived from Peyer's patches after kefir solid fraction administration was also rapidly controlled by the increase of the regulatory IL‐10 (Vinderola, Perdigon, et al., 2006). Kefiran is an exopolysaccharide produced by *L. kefiranofaciens* during fermentation. Kefiran feeding has also been reported to cause increased levels of IgA+B cells, as well as IL‐6, IL‐10, and IL‐12 in the lamina propria of the small intestine in mice (Vinderola, Perdigón, Duarte, Farnworth, & Matar, 2006). A previous study conducted by some investigators from our group on the DSS‐induced colitis model, demonstrated that 5 ml kefir administration once a day reduced the disease activity index, histologic colitis scores, and the increase of TNF alpha (Senol et al., 2015).

The results of the current study showed that an average daily intake of 10 ml kefir per day prevented a decrease in body weight, reduced anorexia, and the incidence of bloody stools and diarrhea induced by TNBS. In addition, 10 ml of kefir attenuated an increase of the colon weight/length ratio and the macroscopic colonic damage caused by TNBS. In contrast, 30 ml kefir administration aggravated TNBS‐induced anorexia, diarrhea, and bloody stools and increased the colonic weight/height ratio and macroscopic colonic damage.

We could not find any study on the dose‐dependent effect of kefir on murine or human colitis in a literature search. To date, many experimental colitis studies involved tests of large numbers of bacterial strains, but few researchers have examined the effective bacterial dose. Pan et al. reported that a high dose of *Lactobacillus paracasei* subsp. *Paracasei* LC‐01 (10^8^–10^10^ cfu/day) attenuated dextran sulfate sodium (DSS)‐induced colitis in BALB/c mice, while no significant effect was observed with a low dose of LC‐01 (Pan et al., 2014). Another study showed that *Lactobacillus crispatus* M247 ameliorates the outcome of DSS colitis in a dose‐dependent manner, reducing colonic MPO activity and body weight loss in 10^8^ and 10^6^ bacteria‐treated mice. The administration of 10^4^ *L. crispatus* M247 did not have a significant effect on colitis outcome (Castagliuolo et al., 2005). In a TNBS rat colitis model, *Escherichia coli* Nissle 1917, at low doses of the strain (10^7^cfu/day), has been demonstrated to reduce the disease activity index, colonic MPO activity, and TNF‐α levels and to increase IL‐10 expression (Sha et al., 2014). However, the study also indicated that the pre‐administration of *Escherichia coli* Nissle 1917 at a dose of 10^9^cfu/day may deteriorate the colitis (Sha et al., 2014).

Chen et al. (2013) also demonstrated that the therapeutic effect of *Lactobacillus acidophilus* on DSS‐induced colitis in BALB/c mice did not increase in a concentration‐dependent manner, but revealed that a moderate‐dose concentration (10^6^cfu/10 g) provided the most alleviation of symptoms, as evidenced by the significant reductions in disease activity index and tissue damage scores (Chen et al., 2013). In addition, the authors found that the number of *Lactobacilli* detected in specific lesions did not increase in conjunction with increased concentrations of the administered *L. acidophilus*. Upon this, the authors hypothesized that interactions between probiotic *L. acidophilus* and local lactobacilli in the intestine (at low concentrations) may serve to promote one another mutually; however, if the probiotic dose is too high, an imbalance between the different lactobacillus sp. may occur, and this may disrupt the mutual promotion and may reduce the beneficial *Lactobacilli* (Chen et al., 2013). This hypothesis may explain why different kefir doses produce opposite outcomes.

Kefir administration has been reported to increase the fecal populations of bifidobacteria and lactobacilli (Liu, Wang, Chen, Yueh, & Lin, 2006) and the number of lactic acid bacteria in the bowel mucosa (Marquina et al., 2002). It is well known that different bacterial strains have different properties, and the effect of a strain is specific for immunomodulatory potential (Zheng et al., 2014). In our opinion, another mechanism explaining the detrimental effect of a dose of kefir that is too high may be its further promotion of the potentially harmful local strains at higher doses.

In our study, the administration of 30 ml kefir per day also did not cause diarrhea, bloody stools, or macroscopic colonic damage in healthy rats. However, a histologic examination of the colon of these rats revealed edema in the lamina propria and submucosa. In a previous study by some researchers in our group, some probiotic strains used in high doses have also been shown to cause lamina propria edema in healthy rats (not published yet). We also found that the average daily chow consumed in the kefir30 control group was lower than in the colitis group. This may be due to the fact that kefir replaces chow as a nutrient, but this may also be associated with anorexia caused by edema in the colon mucosa, as shown histologically. A daily dose of 30 ml of kefir corresponds to the total daily water consumption of a rat, and it is obvious that the dose level is too high.

The fact that kefir has not been used in much more different concentrations is a lack of this study. Nevertheless, these results implicate that even in healthy people, the probiotic dose is important.

## 5 CONCLUSION

Kefir in a daily dose of 10 ml relieves clinical findings and colonic macroscopic damage in TNBS‐induced colitis in rats, but a dose of 30 ml per day exacerbates colitis. These results indicate that kefir may be a useful agent in the treatment of IBD. However, careful dose selection appears crucial in providing beneficial outcomes in clinical trials with kefir in IBD.

## CONFLICT OF INTEREST

The authors declare that they do not have any conflict of interest.

## ETHICAL APPROVAL

The study was carried out at Experimental Research and Pathology Laboratories, Medical School of Suleyman Demirel University. Approval from the Ethical Committee of the Medical School of Suleyman Demirel University was obtained (Decision Date: 08.11.2006, 08/10), and all animals received care in compliance with the “Principles of Laboratory Animal Care” (www.nap.edu/catalog/5140.html).
